# Risk Factors and a Scoring System to Predict ARDS in Patients with COVID-19 Pneumonia in Korea: A Multicenter Cohort Study

**DOI:** 10.1155/2021/8821697

**Published:** 2021-04-09

**Authors:** Jun-Won Seo, Seong Eun Kim, Eun Young Choi, Kyung Soo Hong, Tae Hoon Oh, Uh. Jin Kim, Seung-Ji Kang, Kyung-Hwa Park, Sook-In Jung, Da Young Kim, Na Ra Yun, Dong-Min Kim, Hwa Pyung Kim, Jian Hur, Hee-Chang Jang

**Affiliations:** ^1^Department of Internal Medicine, College of Medicine, Chosun University, Gwangju, Republic of Korea; ^2^Department of Infectious Diseases, Chonnam National University Hospital, Gwangju, Republic of Korea; ^3^Department of Internal Medicine, Yeungnam University Medical Center, Daegu, Republic of Korea; ^4^DEEPNOID, Seoul, Republic of Korea

## Abstract

Predictive studies of acute respiratory distress syndrome (ARDS) in patients with coronavirus disease 2019 (COVID-19) are limited. In this study, the predictors of ARDS were investigated and a score that can predict progression to ARDS in patients with COVID-19 pneumonia was developed. All patients who were diagnosed with COVID-19 pneumonia between February 1, 2020, and May 15, 2020, at five university hospitals in Korea were enrolled. Their demographic, clinical, and epidemiological characteristics and the outcomes were collected using the World Health Organization COVID-19 Case Report Form. A logistic regression analysis was performed to determine the predictors for ARDS. The receiver operating characteristic (ROC) curves were constructed for the scoring model. Of the 166 patients with COVID-19 pneumonia, 37 (22.3%) patients developed ARDS. The areas under the curves for the infiltration on a chest X-ray, C-reactive protein, neutrophil/lymphocyte ratio, and age, for prediction of ARDS were 0.91, 0.90, 0.87, and 0.80, respectively (all *P* < 0.001). The COVID-19 ARDS Prediction Score (CAPS) was constructed using age (≥60 years old), C-reactive protein (≥5 mg/dL), and the infiltration on a chest X-ray (≥22%), with each predictor allocated 1 point. The area under the curve of COVID-19 ARDS prediction score (CAPS) for prediction of ARDS was 0.90 (95% CI 0.86–0.95; *P* < 0.001). It provided 100% sensitivity and 75% specificity when the CAPS score cutoff value was 2 points. CAPS, which consists of age, C-reactive protein, and the area of infiltration on a chest X-ray, was predictive of the development of ARDS in patients with COVID-19 pneumonia.

## 1. Introduction

The coronavirus disease 2019 (COVID-19) pandemic is currently ongoing. Since December 2019, more than 97,000,000 patients have been diagnosed with COVID-19, and the associated mortality rate is about 2% [1]. Several studies evaluating epidemiology, clinical manifestation, risk factors, treatment, and outcomes of patients with COVID-19 have been published, and these studies have shown that the presentation of COVID-19 varies from asymptomatic infection to severe viral pneumonia with acute respiratory distress syndrome (ARDS) in humans [2, 3].

Early detection of the likelihood of worsening to ARDS in patients with COVID-19 pneumonia will help to appropriately identify and classify those who need to be referred to tertiary centers among those requiring simple conservative treatment. However, few studies have evaluated models to predict which patients are likely to develop severe pneumonia such as ARDS. An effective and simple screening tool that can predict the occurrence of ARDS is urgently needed. For that reason, we developed the COVID-19 ARDS Prediction Score (CAPS) that can help to screen patients who are likely to develop into severe respiratory distress in clinical settings.

## 2. Patients and Methods

### 2.1. Ethics

The study was approved by the institutional review boards at Yeungnam University Hospital (IRB No. 2020-03-100), Chonnam National University Hospital (IRB No. CNUH-2020-039), and Chosun University Hospital (CHOSUN 2020-04-003-002).

### 2.2. Study Design and Patients

This retrospective cohort study included all adult patients (≥18 years old) with COVID-19 pneumonia between February 1, 2020, and May 15, 2020, from five hospitals: Yeungnam University Hospital (Daegu, Korea), Chonnam National University Hospital, Chonnam National University Hwasun Hospital, Chonnam National University Bitgoeul Hospital, and Chosun University Hospital (Gwangju, Korea). We evaluated a total of 238 patients with laboratory-confirmed COVID-19. The aim of this study was to identify risk factors that predict ARDS in COVID-19 patients with pneumonia. We excluded the patients with mild disease who had no pneumonic infiltration on chest radiogram or chest computed tomography. Finally, 166 of 238 patients had pneumonia and were enrolled in this study.

Infectious disease physicians in each hospital collected and reviewed the data. Demographic, clinical characteristics, and outcomes were extracted from patients' electronic medical records. Symptoms, infiltration on a chest X-ray, and laboratory findings at the initial diagnosis of pneumonia were collected and used for further analyses. The clinical outcomes were followed up until June 15, 2020.

### 2.3. Definitions

The diagnosis of COVID-19 was made by detection of positive SARS-CoV-2 from respiratory specimens using real-time reverse transcription-polymerase chain reaction (RT-PCR), performed with a kit with a sensitivity and specificity of 95% and 97%, retrospectively (BioCore 2019-nCoV Real-Time PCR Kit, Kogene Biotech, Inc., Seoul, Republic of Korea).

Pulmonary infiltration was classified as patchy, confluent, or nodular, and unilateral or bilateral, by at least two physicians in each hospital. The area of pulmonary infiltration was analyzed by using DEEP:PHI (medical AI software; DEEPNOID, Seoul, Republic of Korea) which is an open platform that supports medical imaging artificial intelligence (AI) model research efficiently.

Oxygen was supplied to patients with oxygen saturation less than 93% in room air. The definition of ARDS is a partial pressure of arterial oxygen (PaO_2_)/percentage of inspired oxygen (FiO_2_) of <300 mmHg [4].

### 2.4. Statistical Analyses

The Kolmogorov-Smirnov test was used to test for normality in continuous variables. Continuous variables were expressed as means ± standard deviation, and the Student *t*-test was used to compare statistical differences, if the variables follow a normal distribution. Continuous variables were expressed as median (interquartile range), and the Mann–Whitney *U* test was used, if the variables follow a nonnormal distribution. Categorical variables were expressed as numbers and percentages and Pearson's chi-squared test or Fisher's exact test was used for comparisons. The receiver operating characteristic (ROC) curve and Youden's index *J* (*J* = sensitivity + specificity − 1) were used to select the optimal cutoff value indicating ARDS. We selected the variables that had an area under the ROC (AUROC) ≥ 0.80 and converted them into categorical variables using cutoff values for the COVID-19 ARDS Prediction Score (CAPS) calculation. Multiple logistic regression analysis was used to determine the predictive factors for the development of ARDS. A two-sided *P* ≤ 0.05 was considered statistically significant and no adjustment was made for multiplicity. The statistical analyses were performed with SPSS ver. 26.0 (IBM, Armonk, NY, USA).

## 3. Results

Clinical characteristics and outcomes of patients are shown in Table 1. A total of 166 patients were identified: 129 (77.7%) patients had pneumonia without ARDS and 37 (22.3%) patients developed ARDS. Older age; presence of comorbidity including hypertension, diabetes mellitus, chronic kidney disease, neurologic disorders, and dementia; presence of dyspnea or fever; and the absence of sore throat, myalgia, or headache were associated with the development of ARDS in the univariate analysis (*P* < 0.05, each; Table 1). Greater levels of lung infiltration on a chest X-ray; leukocytosis; neutrophilia; lymphopenia; thrombocytopenia; and elevated serum aspartate transaminase, bilirubin, glucose, blood urea nitrogen, C-reactive protein (CRP), lactate dehydrogenase, and procalcitonin were significantly associated with COVID-19 pneumonia combined with ARDS rather than those without ARDS (*P* < 0.05, each). COVID-19 pneumonia with ARDS was significantly associated with corticosteroid treatment (Table 1). The mortality rates of patients with COVID-19 pneumonia with ARDS and without ARDS were 43% and 2%, respectively (*P* < 0.001).

AUROC was obtained for the factors that showed significant differences between the patients with COVID-19 pneumonia with ARDS and those without ARDS. Age, area of pulmonary infiltration on a chest X-ray, C-reactive protein, and neutrophil/lymphocyte count showed AUROC values ≥ 0.80 (Table 2). Multiple logistic regression analysis showed that older age (age ≥ 60; odds ratio: 4.6; *P* = 0.035), area of pulmonary infiltration on a chest X-ray (pulmonary infiltration ≥ 22%; odds ratio: 3.5; *P* = 0.023), and elevated CRP (CRP ≥ 5 mg/dL, odds ratio: 10.7; *P* = 0.007) were independent risk factors for prediction of ARDS in patients with COVID-19. The N/L ratio (*P* = 0.143) was excluded because it showed no statistical significance (Table 3) and no additional increase in AUROC when it was included.

**Figure 1:**
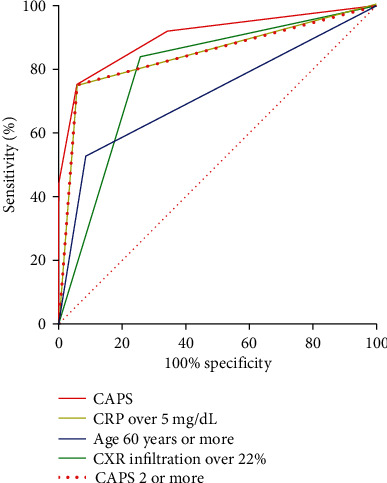
ROC curves of age, infiltration on chest X-ray, C-reactive protein, and COVID-19 ARDS Prediction Score. Acronyms: CAPS = Coronavirus Disease 2019 Acute Respiratory Distress Syndrome Prediction Score; CXR = chest X ray; CRP = C-reactive protein.

Based on the values and regression coefficients of these risk factors, we created a clinical prediction model to predict which of the patients with COVID-19 are at risk of ARDS using scoring points (Table 4). The following variables were scored to develop CAPS: age (≥60 years old), CRP (≥5 mg/dL), and pulmonary infiltration (≥22%) on a chest X-ray (1 point each). The combination of these three parameters created scores ranging from 0 to 3. A COVID-19 ARDS Prediction Score model was constructed as CAPS = (1 × age over 60) + (1 × CRP over 5) + (1 × pulmonary infiltration on a chest X − ray over 22%). We used this simple scoring system because there was no significant difference in the results when we used a more detailed weighting system. On the ROC curve obtained for the model, the optimal cutoff score of ≥2 had 94% sensitivity and 75% specificity for the occurrence of ARDS, with a ROC area under the curve of 0.85 (Tables 5 and 6 and Figure 1).

## 4. Discussion

The emergence of COVID-19 caused a global pandemic, and most deaths are caused by pulmonary complications such as ARDS. Recently, several antiviral therapies including remdesivir [5] and dexamethasone [6] have shown beneficial effects on COVID-19 in randomized clinical trials. However, the benefits of these treatments were shown to be superior to placebo by shortening the time to recovery in some patients with mild to moderate symptoms, and not in severely and critically ill patients. For this reason, antiviral treatment should be initiated before the progression to severe disease. However, the number of therapeutic agents is limited, and it is unnecessary to treat all of the mild COVID-19 cases with antiviral agents. In this situation, the systematic and reliable prediction system will be beneficial for screening patients who are expected to develop severe disease, such as ARDS, requiring transfer to a specialized medical center for appropriate treatment.

Several studies have developed models that predict the diagnosis of COVID-19 and risk factors for disease severity [7–12]. Wu et al. reported that old age and fever are risk factors associated with ARDS and death in patients with COVID-19 pneumonia [13], and Zhang et al. reported that D-dimer could be a predictive factor for in-hospital mortality in patients with COVID-19 [14]. However, there are few studies of prediction models for the occurrence of ARDS or ICU admission. Liang et al. reported that chest radiography, age, hemoptysis, dyspnea, unconsciousness, number of comorbidities, cancer history, neutrophil-to-lymphocyte ratio, lactate dehydrogenase, and direct bilirubin were associated with an increased risk of ICU requirement or death, and a scoring system using above variables showed an AUC of 0.88 [15].

In this cohort study, we showed that older age, initial pulmonary infiltration on a chest X-ray, and CRP were independent predictors of ARDS occurrence for patients with COVID-19 pneumonia. In our study, dyspnea was not presented as an important predictor of ARDS, as this is a categorical variable (all or nothing). Instead, we introduced respiratory rate for predicting ARDS. However, respiratory rate showed inferiority for predicting ARDS compared to dyspnea (AUROC: 0.67; cutoff value: 22/min; sensitivity: 46%; specificity: 89%). This may be due to clinical evaluation at the time of diagnosing COVID-19 pneumonia, as subjective dyspnea may not have been present at that early stage of the disease.

Our prediction model has merit because we included detailed chest X-ray findings, which are used by most clinicians to estimate the severity and are more objective than the symptoms of patients. C-reactive protein, which is a widely used inflammatory marker for patients with infectious diseases, was more predictive of ARDS in this study than other laboratory values, which is consistent with a previous study [16]. The neutrophil-lymphocyte count ratio, neutrophilia, and lymphopenia were also associated with ARDS in this study, which is consistent with previous studies [17]. However, these parameters did not provide additional diagnostic value to the model consisting of age, C-reactive protein, and chest X-ray findings.

This study had several limitations. First of all, it may possess selection bias due to the retrospective design, even though it was a multicenter cohort study. Second, this study was conducted in a single country with a single genetic background. Third, this study had the absence of a validation cohort. Fourth, the symptoms and laboratory result used in this study were not standardized, so they could have confounding bias. For these reasons, further studies are needed to validate this score and allow generalization to various countries and races.

In conclusion, CAPS ≥ 2 points, which takes age (≥60), CRP (≥5 mg/dL), and area of pulmonary infiltration on a chest X-ray (≥22%) into consideration, could effectively predict the occurrence of ARDS in patients with COVID-19 pneumonia. Therefore, CAPS could be an early and helpful prediction model to improve the outcome of patients with COVID-19.

## Figures and Tables

**Table 1:** Demographic, clinical characteristics, treatment, and outcome of 166 patients with COVID-19 pneumonia.

Characteristics	Patients without ARDS (*n* = 129)	Patients with ARDS (*n* = 37)	*P* value
Demographic data			
Male sex	56 (43)	22 (60)	0.095
Age (year)^a^	56 ± 17	72 ± 11	<0.001
20-59^c^	67 (96)	3 (4)	<0.001
≥60^c^	62 (65)	34 (35)	
Body mass index (kg/m^2^)^a^	23.7 ± 3.3	25.2 ± 3.1	0.029

Comorbidity			
Hypertension^c^	38 (30)	21 (57)	0.002
Use of ACEi or ARB^c^	19 (15)	7 (19)	0.420
Diabetes mellitus^c^	19 (15)	13 (35)	0.006
Chronic cardiac disease^d^	10 (8)	1 (3)	0.459
Chronic pulmonary disease^d^	4 (3)	4 (11)	0.075
Chronic kidney disease^d^	2 (2)	4 (11)	0.023
Neurologic disorder^c^	5 (4)	6 (16)	0.008
Dementia^d^	3 (2)	5 (14)	0.014

Symptom at presentation			
Asymptomatic^d^	5 (4)	0 (0)	0.588
Respiratory symptoms			
Cough^c^	79 (61)	22 (60)	0.804
Sputum^c^	60 (47)	18 (49)	0.849
Rhinorrhea^d^	11 (9)	2 (5)	0.734
Sore throat^d^	26 (20)	2 (5)	0.044
Dyspnea^c^	47 (36)	26 (70)	<0.001
Nonrespiratory symptoms			
Fever^c^	82 (64)	31 (84)	0.023
Myalgia^c^	50 (39)	7 (19)	0.022
Headache^d^	47 (36)	4 (11)	0.002
Altered consciousness^d^	2 (2)	3 (8)	0.074
Nausea or vomiting^c^	10 (8)	5 (14)	0.295
Diarrhea^c^	24 (19)	5 (14)	0.450

Lung infiltration on chest X-ray at presentation			
No infiltration	78 (60)	2 (5)	<0.001
Infiltration on chest X-ray (%)^a^	8 ± 15	41 ± 27	<0.001

Laboratory finding at presentation			
White blood cell count (/mm^3^)^b^	5500 (4505, 7280)	7400 (4945, 9805)	0.001
Neutrophil count (/mm^3^)^b^	3582 (2503, 4788)	6433 (3719, 7903)	<0.001
Neutrophil %^a^	64 ± 12	82 ± 10	<0.001
Lymphocyte count (/mm^3^)^a^	1470 ± 658	803 ± 419	<0.001
Lymphocyte %^a^	26 ± 11	12 ± 7	<0.001
Platelet (×10^3^/mm^3^)^a^	246 ± 101	176 ± 63	<0.001
Aspartate transaminase (U/L)^b,e^	31 (24, 44)	48 (37, 68)	<0.001
Alanine transaminase (U/L)^b,e^	23 (15, 38)	25 (14, 44)	0.933
Total bilirubin (mg/dL)^b,f^	0.69 (0.51, 0.96)	0.84 (0.70, 1.31)	0.009
Glucose (mg/dL)^b,g^	116 (97, 134)	139 (120, 171)	<0.001
Blood urea nitrogen (mg/dL)^b,h^	13 (10, 16)	20 (12, 28)	0.001
Creatinine (mg/dL)^b,f^	0.76 (0.63, 0.95)	0.89 (0.69, 1.25)	0.023
Lactate (mg/dL)^b,i^	1.9 (1.2, 2.5)	1.6 (1.3, 2.2)	0.689
C-reactive protein (mg/L)^b,j^	1.2 (0.1, 5.4)	16.4 (7.8, 21.9)	<0.001
Lactate dehydrogenase (U/L)^b,k^	501 (400, 678)	811 (557, 1164)	<0.001
Procalcitonin^b,l^	0.04 (0.02, 0.08)	0.37 (0.12, 0.81)	<0.001

Outcome			
O_2_ supply during the hospital stay^d^	34 (26)	37 (100)	<0.001
Mechanical ventilation^d^	20 (14)	17 (94)	<0.001
Death^d^	3 (2)	16 (43)	<0.001

Categorical variables were expressed as number (%) and were compared using Pearson's chi-squared test or Fisher's exact test. Pearson's chi-square test was applied if all expected frequencies have 5 or more counts, and Fisher's exact test was applied if expected frequency has less than 5. Kolmogorov-Smirnov's test was used for normality in continuous variables. (a) Expressed as means ± standard deviation and compared by Student's *t*-test. (b) Expressed as median (interquartile range) and compared by Mann–Whitney *U* test. (c) Fisher's exact test was used to evaluate statistical significance. (d) Pearson's chi-squared test was used to evaluate statistical significance. (e) Measured in 127 non-ARDS and 37 ARDS patients. (f) Measured in 123 non-ARDS and 37 ARDS patients. (g) Measured in 120 non-ARDS and 36 ARDS patients. (h) Measured in 128 non-ARDS and 37 ARDS patients. (i) Measured in 48 non-ARDS and 34 ARDS patients. (j) Measured in 125 non-ARDS and 35 ARDS patients. (k) Measured in 122 non-ARDS and 35 ARDS patients. (l) Measured in 117 non-ARDS and 33 ARDS patients. Acronyms: ARDS = acute respiratory distress syndrome; ACEi = angiotensin converting enzyme inhibitor; ARB = angiotensin receptor blocker.

**Table 2:** Area under the ROC curve and optimal cutoff value of variables indicating acute respiratory distress syndrome in COVID-19.

	AUROC	*P* value	95% CI	Cutoff value	Sensitivity (%)	Specificity (%)	PPV (%)	NPV (%)
*Continuous variables*								
C-reactive protein (mg/dL)	0.90	<0.001	0.85–0.94	5.0	94	74	51	98
Infiltration on chest X ray (%)	0.87	<0.001	0.81–0.94	22	74	84	57	91
Neutrophil/lymphocyte ratio	0.87	<0.001	0.80–0.93	3.55	90	71	47	97
Lymphocyte count (/mm^3^)	0.81	<0.001	0.74–0.88	1021	70	74	44	90
Neutrophil count (/mm^3^)	0.77	<0.001	0.68–0.86	5373	67	82	43	90
Age (year)	0.80	<0.001	0.72–0.87	60	92	53	35	96
Lactate dehydrogenase (U/L)	0.74	<0.001	0.64–0.85	600	71	65	37	89
Aspartate transaminase (U/L)	0.73	<0.001	0.64–0.83	41	70	71	41	89
Platelet (×10^3^/mm^3^)	0.72	<0.001	0.63–0.81	204	57	60	29	83
Glucose (mg/dL)	0.72	<0.001	0.63–0.81	125	75	66	40	90
Blood urea nitrogen (mg/dL)	0.68	0.001	0.56–0.79	14.6	62	63	33	85
Body mass index (kg/m^2^)	0.65	0.010	0.55–0.75	24.2	66	67	32	88
Creatinine (mg/dL)	0.62	0.023	0.51–0.73	0.83	60	60	30	84

*Categorical variables*								
Dyspnea	0.67	0.002	0.57–0.77		70	64	36	88
Hypertension	0.64	0.011	0.53–0.74		57	71	36	85
Headache	0.63	0.015	0.54–0.73		89	37	8	71
Diabetes mellitus	0.60	0.059	0.49–0.71		35	85	41	82
Fever	0.60	0.068	0.50–0.70		84	36	27	88
Myalgia	0.60	0.059	0.50–0.70		81	39	12	72
Sore throat	0.58	0.164	0.48–0.67		95	21	7	74
Dementia	0.56	0.300	0.45–0.67		14	98	63	80
Chronic kidney disease	0.55	0.391	0.44–0.66		11	98	67	79

Acronyms: AUROC = area under the receiver operating characteristic curve; PPV = positive predictive value; NPV = negative predictive value.

**Table 3:** Risk factors for ARDS in 160 patients with COVID-19 in multivariate analysis.

Characteristics	Univariate analysis					Multivariate analysis		
	Non-ARDS (*n* = 125)	ARDS (*n* = 35)	Odds ratio	95% CI	*P* value	Adjusted odds ratio	95% CI	*P* value
Infiltration on chest X − ray ≥ 22%	20 (16)	26 (74)	15.2	6.2–37.0	<0.001^∗^	3.5	1.18–10.13	0.023^∗^
C − reactive protein ≥ 5 mg/dL	32 (26)	33 (94)	47.6	10.9–200	<0.001^∗^	10.7	1.92–59.86	0.007^∗^
Age ≥ 60 years	59 (47)	32 (91)	11.9	3.5–41.7	<0.001^∗^	4.6	1.21–20.18	0.035^∗^
Neutrophil/lymphocyte ratio ≥ 3.5	36 (29)	32 (91)	26.3	7.6–90.9	<0.001^∗^	3.2	0.61–13.72	0.181

^∗^Statistically significant (*P* ≤ 0.05). Acronyms: ARDS = acute respiratory distress syndrome; CI = confidence interval.

**Table 4:** Area under the ROC curve of COVID-19 ARDS Prediction Score.

	Point	AUROC	95% CI	*P* value
Covid-19 ARDS Prediction Score	**0-3**	**0.90**	**0.85–0.95**	**<0.001**
C − reactive protein ≥ 5 mg/dL	1	0.84	0.78–0.91	<0.001
Infiltration on chest X − ray ≥ 22%	1	0.79	0.77–0.91	<0.001
Age ≥ 60 years	1	0.73	0.64–0.80	<0.001

Acronyms: AUROC = area under the receiver operating characteristic curve; CI = confidence interval; ARDS = acute respiratory distress syndrome.

**Table 5:** Optimal cutoff value for COVID-19 ARDS Prediction Score (CAPS).

	Cutoff value	AUROC	95% CI	*P* value	Sensitivity (%)	Specificity (%)	PPV (%) (^∗^95% CI)	NPV (%) (^∗^95% CI)
CAPS	2	0.85	0.78–0.91	<0.001	94	75	52 (47–56)	98 (97–99)

Acronyms: AUROC = area under the receiver operating characteristic curve; CI = confidence interval; PPV = positive predictive value; NPV = negative predictive value. ^∗^Confidence interval of PPV and NPV was calculated using the Clopper-Pearson exact method.

**Table 6:** Distribution of 160 patients according to the COVID-19 ARDS Prediction Score (CAPS).

Score	Non-ARDS (*n* = 125)	ARDS (*n* = 35)	PPV (%)	NPV (%)
0	55 (44)	0 (0)		
1	39 (31)	2 (6)		
2	21 (17)	10 (29)		
3	10 (8)	23 (66)		
<2	94 (75)	2 (6)	2	98
≥2	31 (25)	33 (94)	52	48

Expressed as number (%). Acronyms: ARDS = acute respiratory distress syndrome; CI = confidence interval; PPV = positive predictive value; NPV = negative predictive value.

## Data Availability

All the information supporting our conclusions and relevant references are included in the manuscript. Corresponding author HCJ can be contacted for more information.
